# Supplementary material for: Role of PI3K-AKT Pathway in Ultraviolet Ray and Hydrogen Peroxide-Induced Oxidative Damage and Its Repair by Grain Ferments
Source: Foods. 2023 Feb 13;12(4):806. doi: 10.3390/foods12040806 (PMC9957031; doi:10.3390/foods12040806)
Supplement: Supplementary file 1 [file foods-12-00806-s001.zip › foods-2123019-supplementary.pdf]

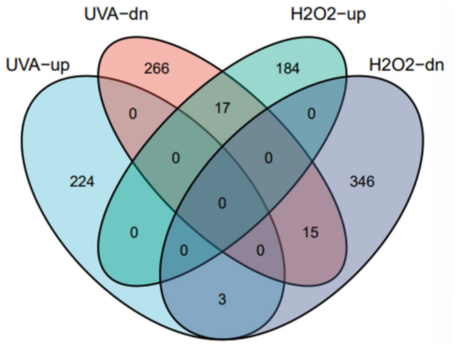

Figure S1. Venn diagram of the two models. UVA-up: up-regulated DEGs in UVA-induced model; UVA-dn: down-regulated DEGs in UVA-induced model; H_2O_2-up: up-regulated DEGs in H_2O_2-induced model; H_2O_2-dn: down-regulated DEGs in H_2O_2-induced model.

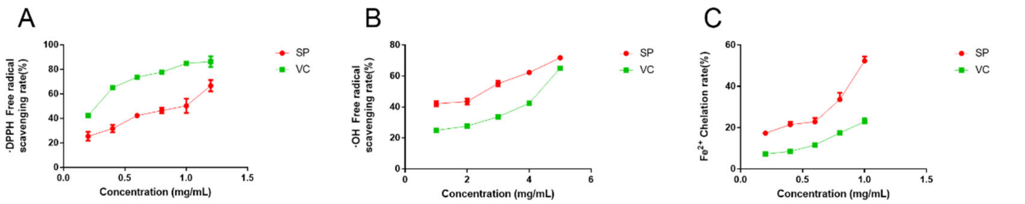

Figure S2. Determination of antioxidant capacities of *S. commune* polysaccharide (SP) and VC in vitro. A, DPPH• scavenging effects; B, •OH free radicals scavenging effects; C, Fe^{2+} chelating abilities. VC, namely ascorbic acid, was chosen as the positive control. Results were expressed as the mean ±SD(n=3).

Table S1. UVA-induced model.

Categories	Term	Count	Pvalue
**Annotation Cluster 1 cell adhesion**			
**Enrichment Score: 5.98**			
GOTERM_BP_DIRECT	GO:0007229~integrin-mediated signaling pathway	10	1.75E-10
GOTERM_BP_DIRECT	GO:0033627~cell adhesion mediated by integrin	7	4.41E-09
GOTERM_BP_DIRECT	GO:0007160~cell-matrix adhesion	8	1.06E-07
GOTERM_BP_DIRECT	GO:0031589~cell-substrate adhesion	5	7.15E-07
GOTERM_BP_DIRECT	GO:0046718~viral entry into host cell	7	1.51E-06
GOTERM_BP_DIRECT	GO:0034113~heterotypic cell-cell adhesion	5	3.42E-06
GOTERM_BP_DIRECT	GO:0098609~cell-cell adhesion	8	5.43E-06
GOTERM_BP_DIRECT	GO:0033631~cell-cell adhesion mediated by integrin	3	1.90E-04
GOTERM_BP_DIRECT	GO:0007159~leukocyte cell-cell adhesion	3	5.21E-03
GOTERM_BP_DIRECT	GO:0045995~regulation of embryonic development	5	8.81E-07

GOTERM_BP_DIRECT	GO:0030155~regulation of cell adhesion	6	2.25E-06
GOTERM_BP_DIRECT	GO:0030334~regulation of cell migration	6	4.00E-05
**Annotation Cluster 2 regulation of MAP kinase activity and MAPK cascade**			
**Enrichment Score: 5.61**			
GOTERM_BP_DIRECT	GO:0043406~positive regulation of MAP kinase activity	8	1.48E-08
GOTERM_BP_DIRECT	GO:0033674~positive regulation of kinase activity	6	6.50E-06
GOTERM_BP_DIRECT	GO:0007169~transmembrane receptor protein tyrosine kinase signaling pathway	7	8.02E-06
GOTERM_BP_DIRECT	GO:0046777~protein autophosphorylation	7	4.65E-05
GOTERM_BP_DIRECT	GO:0007275~multicellular organism development	6	1.11E-03
GOTERM_BP_DIRECT	GO:0018108~peptidyl-tyrosine phosphorylation	5	1.32E-03
GOTERM_BP_DIRECT	GO:0038083~peptidyl-tyrosine autophosphorylation	3	1.88E-03
GOTERM_BP_DIRECT	GO:0043406~positive regulation of MAP kinase activity	8	1.48E-08
GOTERM_BP_DIRECT	GO:0014068~positive regulation of phosphatidylinositol 3-kinase signaling	8	1.77E-08
GOTERM_BP_DIRECT	GO:0043410~positive regulation of MAPK cascade	9	1.03E-07
GOTERM_BP_DIRECT	GO:0010863~positive regulation of phospholipase C activity	3	5.65E-04
GOTERM_BP_DIRECT	GO:0043552~positive regulation of phosphatidylinositol 3-kinase activity	3	5.91E-03
**Annotation Cluster 3 cell proliferation and division**			
**Enrichment Score: 3.79**			
GOTERM_BP_DIRECT	GO:0051897~positive regulation of protein kinase B signaling	7	6.72E-06
GOTERM_BP_DIRECT	GO:0001938~positive regulation of endothelial cell proliferation	6	6.95E-06
GOTERM_BP_DIRECT	GO:0002042~cell migration involved in sprouting angiogenesis	4	5.68E-05
GOTERM_BP_DIRECT	GO:0010595~positive regulation of endothelial cell migration	5	9.96E-05
GOTERM_BP_DIRECT	GO:0045766~positive regulation of angiogenesis	6	2.87E-04
GOTERM_BP_DIRECT	GO:0043536~positive regulation of blood vessel endothelial cell migration	4	7.76E-04
GOTERM_BP_DIRECT	GO:0001934~positive regulation of protein phosphorylation	6	8.43E-04
GOTERM_BP_DIRECT	GO:0070374~positive regulation of ERK1 and ERK2 cascade	5	8.96E-03

GOTERM_BP_DIRECT	GO:0050679~positive regulation of epithelial cell proliferation	5	8.32E-05
GOTERM_BP_DIRECT	GO:0001934~positive regulation of protein phosphorylation	6	8.43E-04
GOTERM_BP_DIRECT	GO:0030324~lung development	4	4.20E-03
GOTERM_BP_DIRECT	GO:0070374~positive regulation of ERK1 and ERK2 cascade	5	8.96E-03
GOTERM_BP_DIRECT	GO:0051781~positive regulation of cell division	3	1.30E-02
GOTERM_BP_DIRECT	GO:0007399~nervous system development	5	4.44E-02
GOTERM_BP_DIRECT	GO:0030154~cell differentiation	5	2.17E-01

Table S2. H_2O_2-induced model

Categories	Term	Count	Pvalue
**Annotation Cluster 1 cell adhesion**			
**Enrichment Score: 11.81**			
GOTERM_BP_DIRECT	GO:0007160~cell-matrix adhesion	13	1.21E-16
GOTERM_BP_DIRECT	GO:0033627~cell adhesion mediated by integrin	10	8.47E-16
GOTERM_BP_DIRECT	GO:0007229~integrin-mediated signaling pathway	12	8.44E-15
GOTERM_BP_DIRECT	GO:0007155~cell adhesion	18	8.96E-14
GOTERM_BP_DIRECT	GO:0098609~cell-cell adhesion	10	2.78E-09
GOTERM_BP_DIRECT	GO:0050900~leukocyte migration	4	6.60E-05
**Annotation Cluster 2**			
**Enrichment Score: 1.93**			
GOTERM_BP_DIRECT	GO:0007275~multicellular organism development	6	3.59E-04
GOTERM_BP_DIRECT	GO:0033674~positive regulation of kinase activity	3	1.80E-02
GOTERM_BP_DIRECT	GO:0018108~peptidyl-tyrosine phosphorylation	3	5.34E-02
GOTERM_BP_DIRECT	GO:0007169~transmembrane receptor protein tyrosine kinase signaling pathway	3	5.34E-02
**Annotation Cluster 3 inflammatory**			
**Enrichment Score: 1.79**			
GOTERM_BP_DIRECT	GO:0032755~positive regulation of interleukin-6 production	4	2.90E-03
GOTERM_BP_DIRECT	GO:0032757~positive regulation of interleukin-8 production	3	1.41E-02
GOTERM_BP_DIRECT	GO:0006954~inflammatory response	4	1.05E-01
